# In Vivo Experimental Endovascular Uses of Cyanoacrylate in Non-Modified Arteries: A Systematic Review

**DOI:** 10.3390/biomedicines9091282

**Published:** 2021-09-21

**Authors:** Kévin Guillen, Pierre-Olivier Comby, Olivier Chevallier, Anne-Virginie Salsac, Romaric Loffroy

**Affiliations:** 1 Department of Vascular and Interventional Radiology, Image-Guided Therapy Center, François-Mitterrand University Hospital, 14 Rue Paul Gaffarel, BP 77908, 21079 Dijon, France; kevin.guillen@chu-dijon.fr (K.G.); olivier.chevallier@chu-dijon.fr (O.C.); 2 Imaging and Artificial Vision (ImViA) Laboratory-EA 7535, University of Bourgogne/Franche-Comté, 9 Avenue Alain Savary, BP 47870, 21078 Dijon, France; pierre-olivier.comby@chu-dijon.fr; 3 Department of Neuroradiology and Emergency Radiology, François-Mitterrand University Hospital, 14 Rue Paul Gaffarel, BP 77908, 21079 Dijon, France; 4 Biomechanics and Bioengineering Laboratory, UMR CNRS 7338, Université de Technologie de Compiègne, 60203 Compiègne, France; anne-virginie.salsac@utc.fr

**Keywords:** cyanoacrylate, NBCA, endovascular, animal studies, swine, rabbit, rat, dog, artery, artery model

## Abstract

Cyanoacrylates were first used for medical purposes during World War II to close skin wounds. Over time, medical applications were developed, specifically in the vascular field. Uses now range from extravascular instillation in vascular grafting to intravascular injection for embolization. These applications were made possible by the conduct of numerous preclinical studies involving a variety of tests and outcome measures, including angiographic and histological criteria. Cyanoacrylates were first harshly criticized by vascular surgeons, chiefly due to their fast and irreversible polymerization. Over the past five years, however, cyanoacrylates have earned an established place in endovascular interventional radiology. Given the irreversible effects of cyanoacrylates, studies in animal models are ethically acceptable only if supported by reliable preliminary data. Many animal studies of cyanoacrylates involved the experimental creation of aneurysms or arteriovenous fistulas, whose treatment by endovascular embolization was then assessed. In clinical practice, however, injection into non-modified arteries may be desirable, for instance, to deprive a tumor of its vascular supply. To help investigators in this field select the animal models and procedures that are most appropriate for their objectives, we have reviewed all published in vivo animal studies that involved the injection of cyanoacrylates into non-modified arteries to discuss their main characteristics and endpoints.

## 1. Introduction

N-butyl cyanoacrylate (NBCA) is generating considerable interest among interventional radiologists given its good efficacy and biological tolerance when used for endovascular embolization. Practices regarding the clinical uses of NBCAs vary across countries. For instance, Glubran^®^ 2 (GEM, Viareggio, Italy) has obtained the European Conformity (EC) mark, whereas Trufill^®^ (Cordis, Miami Lakes, FL, USA) has been approved by the Food and Drug Administration in the US but is not available elsewhere. Histoacryl^®^ is also an NBCA and is available in the USA and Japan but is normally “off-label” in Europe for endovascular purposes in humans, although it has been used for a long time. Bucrylate^®^, or isobutyl 2-cyanoacrylate (IBCA), was used in early studies before it was demonstrated that NBCA had a better safety profile and greater tensile strength. General agreement exists about the usefulness, when performing intra-arterial injections of cyanoacrylates, of adding a radio-opaque agent that delays polymerization and provides angiographic guidance for controlling polymerization kinetics [1].

Preclinical studies are one of the available means of increasing our knowledge of NBCAs and identifying their best clinical applications. The adequate conceptualization of relevant experimental models is needed to apply an evidence-based methodology capable of producing a high level of basic science evidence. Most studies of the in vivo uses of endovascular NBCA focused on aneurysms or arteriovenous malformations (AVMs) [2]. Different models have been developed to mimic different clinical situations. Thus, rete mirabile embolization through the ascending pharyngeal artery in swine serves as a model for the treatment of AVMs [3]. Aneurysms, including aortic aneurysms, have been created in rabbits and swine and in arteriovenous fistulas in dogs [4,5,6,7,8]. However, to our knowledge, no review of cyanoacrylate injection into non-modified arteries in animal models is available. Embolization by injection into non-modified arteries is sometimes needed in clinical practice, for instance, to deprive a tumor of its blood supply before surgical excision, or to stop arterial bleeding via the transfemoral approach [9]. Moreover, knowledge of the behavior of cyanoacrylates in non-modified arteries may prove valuable.

When interest in cyanoacrylates—which have been available for many years—was recently rekindled, studies identified several issues requiring further investigation [10]. The objective of this systematic review of the experimental studies of cyanoacrylates injected into non-modified arteries in animal models was to help investigators choose the in vivo model and embolization site most likely to provide the answer to their research questions based on all the data published to date.

## 2. Materials and Methods

### 2.1. Literature Search Strategy

We performed an exhaustive computerized search of the Medline/PubMed and Web of Science databases, which complement each other. The search period was 1950 to 2020. We used the following keywords in all possible paired combinations separated by the Boolean operator AND: “cyanoacrylate”, “lipiodol”, “endovascular”, “in vivo”, “experimental”, “mice”, “sheep”, “dog”, “rabbit”, and “primate”. We then manually checked the reference lists of the articles that were retrieved.

Study inclusion criteria were as follows: English or French language, original research (conferences, letters, abstracts, and editorials were not included), publication after peer review, use of cyanoacrylate for endovascular arterial embolization, and no experimental change to the embolized artery (i.e., no aneurysms or AVMs were created). The second author (P.O.C.) independently assessed the eligibility of the studies and the reproducibility of the computerized search. Disagreements were resolved by having P.O.C. and R.L. review the article together to reach a consensus.

### 2.2. Outcomes

To our knowledge, no standardized reviewing protocol exists for experimental animal studies. We drew from the Preferred Reporting Items for Systematic Reviews and Meta-Analyses (PRISMA) extension for scoping reviews (PRISMA-ScR), although it was designed for clinical reviews [11,12]. The data from the selected studies were entered into an electronic spreadsheet listing the following characteristics: publication (first author’s name and country, year, URL); primary study objective; species, number of animals used, and number of models; clinical situations the model was designed to mimic; chemical and commercial names of the cyanoacrylate used; any other compounds used, with the ratio relative to cyanoacrylate; name of the embolized vessels and how they were approached; amount injected; size of the introducer and microcatheters; guiding modality; detailed outcomes (e.g., histological and angiographic findings); and follow-up duration and data. Our findings are reported as a narrative review, with references but without comparisons across studies.

## 3. Results

### 3.1. Included Studies

**Figure 1:**
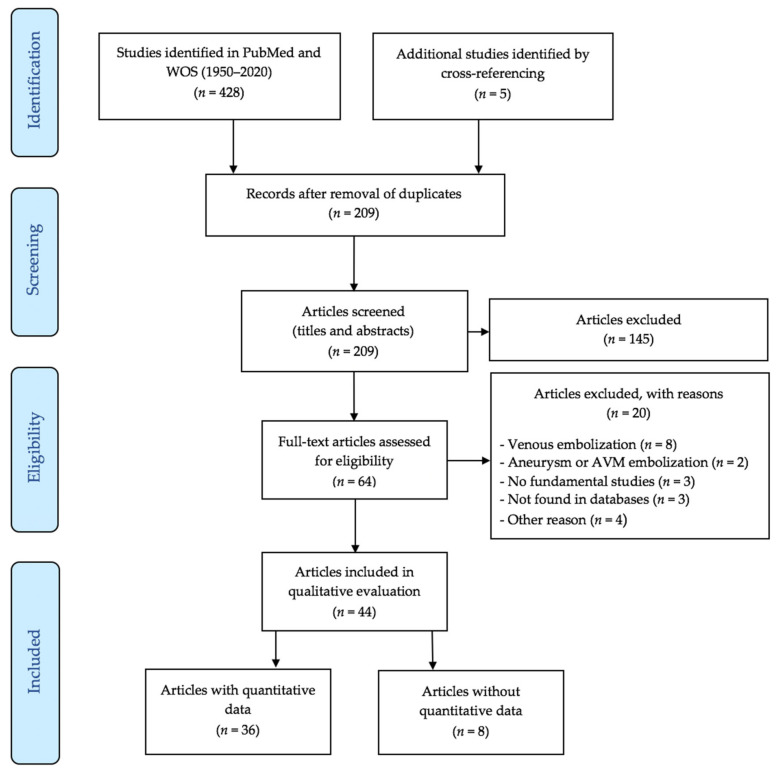
Flow chart. WOS, Web of Science; AVM, arteriovenous malformation.

Our exhaustive search strategy retrieved 428 articles. Figure 1 is the flowchart. After the initial screening of the titles and abstracts, we excluded 141 papers, most of which dealt with the NBCA embolization of experimentally produced AVMs or aneurysms. When the same study involved the endovascular treatment of both prepared and non-modified arteries, only the data regarding the non-modified arteries were considered. The crossmatching of reference lists and review articles identified five additional studies, leading to a total of 64 studies. After the examination of the full texts, we found that 20 articles did not meet our selection criteria; for example, eight of them studied intravenous cyanoacrylate.

**Figure 2:**
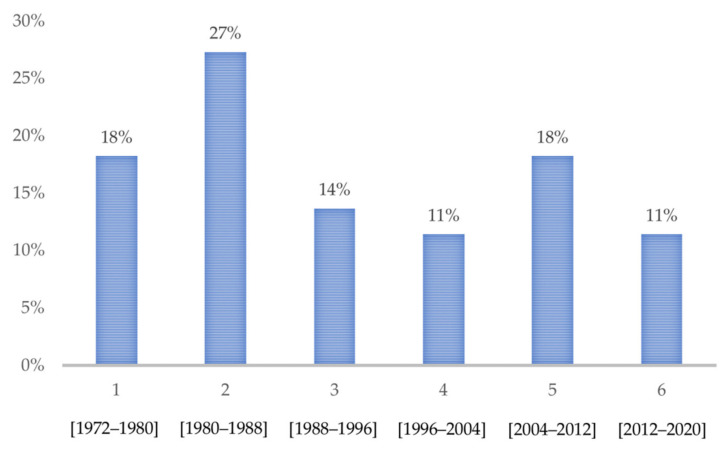
Percentage of studies identified during consecutive 8-year periods from 1972 to 2020.

This left 44 studies for our qualitative analysis of the model characteristics. Their distribution by year of publication is shown in Figure 2. Of the 44 studies, 16 were completed in swine, 17 in dogs, 7 in rabbits, 2 in primates, and 2 in other species. The information needed for a quantitative evaluation of the embolization mixture and procedure characteristics was supplied by 36 of the 44 articles.

### 3.2. General Characteristics

**Figure 3:**
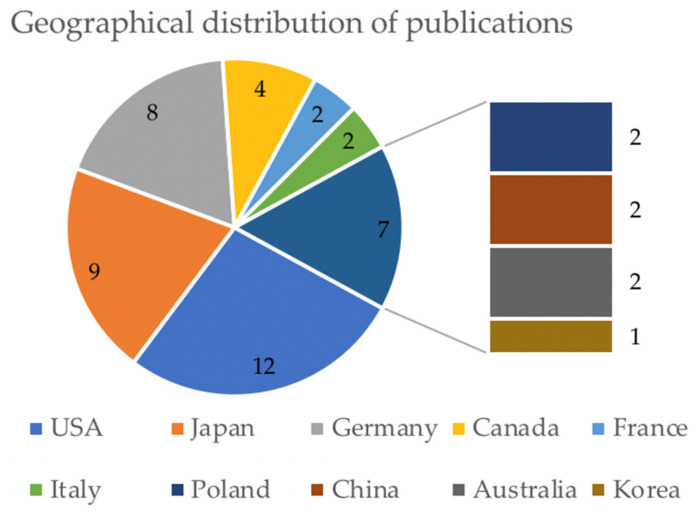
Geographical distribution of the first authors of the included studies (graphic indicates the number of publications of each country).

Most of the publications were by North American authors (USA and Canada), although Japan and China also made substantive contributions (Figure 3). The total number of animals used was 1042, with 454 rats in 4 studies. The main objectives fell into two groups: the evaluation of polymerization kinetics or other characteristics (33 studies, including 11 with Histoacryl^®^ (B/Braun, Tuttlingen, Germany), 8 with Bucrylate^®^, and 1 with Glubran^®^ 2), and the development of models that replicated clinical situations, such as ischemia (six studies, half of which used Histoacryl^®^ and none Glubran^®^ 2). In five other studies, cyanoacrylates were used to document the characteristics of a new tool (e.g., a balloon-microcatheter) or the effects of embolization on the neighboring tissues. Overall, the mean model follow-up was 31 days, the median was 3 days, and the range was 0 to 180 days (with the longest follow-up in mongrel dogs); of the 44 studies, 15 involved euthanasia on the same day as the endovascular intervention.

### 3.3. Procedures

Most of the procedures were completed under fluoroscopic guidance (41/44, 93%) via the transfemoral approach. In the remaining three studies, the microcatheter was inserted directly into the target artery.

Table 1, Table 2 and Table 3 report the main quantitative data in studies of dogs, swine, and rabbits, respectively. Three articles, on primate and rat models, were not considered for the quantitative evaluation, which thus relied on 33 articles [8,13,14,15,16,17,18,19,20,21,22,23,24,25,26,27,28,29,30,31,32,33,34,35,36,37,38,39,40,41,42,43,44,45].

**Figure 4:**
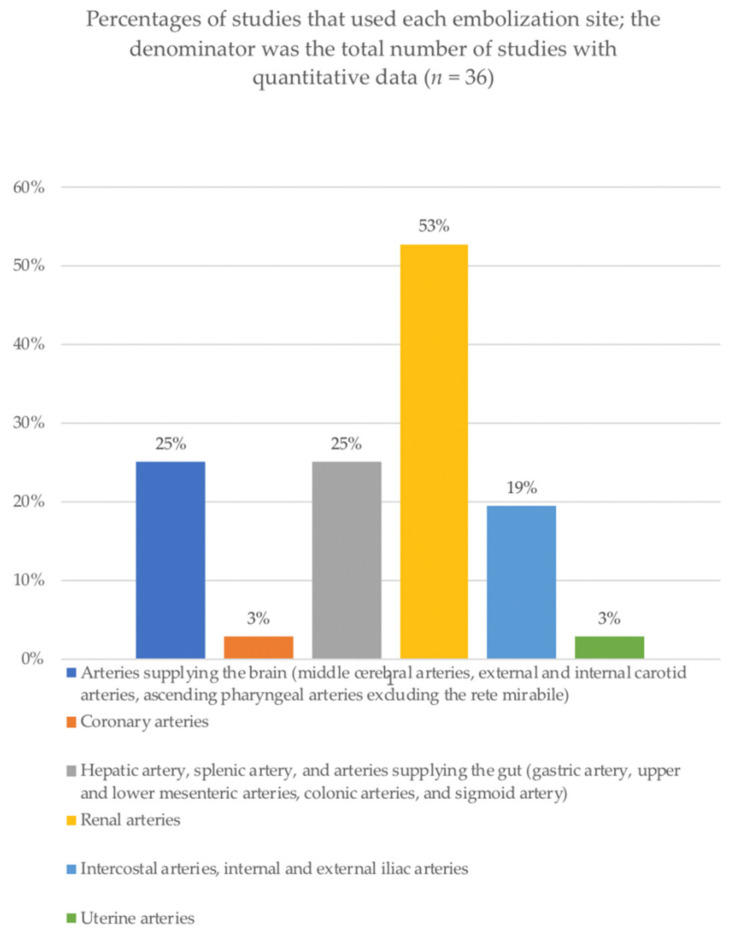
Percentages of studies that used each embolization site; the denominator was the total number of studies with quantitative data (*n* = 36).

The ranges of end-tip external diameters for dog, swine, and rabbit models were 1.0–4.2 French (Fr), 2.0–4.1 Fr, and 1.5–3.6 Fr, respectively. The cyanoacrylate absolute concentrations in the injected substance ranged from 10% to 100%. Figure 4 is a histogram showing the frequencies of arterial sites used for embolization. In 19 (57%) of the 33 studies, the renal artery was embolized. When radio-opaque agents were added to allow real-time control of the embolization while also adjusting the polymerization time, Lipiodol was chosen in 47% and iophendylate in 22% of the cases. Importantly, iophendylate was used in the earliest studies, and Lipiodol was used in the more recent work. Tantalum powder was the third most commonly used contrast agent and had no effect on cyanoacrylate polymerization characteristics.

## 4. Discussion

This review aimed to identify all of the studies that involved cyanoacrylate injection into non-modified arteries of animal models and were published between the early 1970s and now. We did not consider studies of aneurysm embolization as the methods used to produce aneurysms are associated with multiple sources of confounding. More specifically, the need to infuse a heparin sulfate solution and the induced local inflammation of the artery influence the reproducibility of aneurysm models [46]. Furthermore, although successful in vivo animal experiments do not necessarily predict clinical efficacy, they are of interest for developing embolic agents given the physicochemical similarities between experimental animals and humans [17,47].

Animal experimentation has a number of drawbacks. First, sophisticated and costly tools that are appropriate for the species that is used are needed. Moreover, all in vivo imaging studies and interventions require controlled analgesia and monitoring in an accredited laboratory. The relevance of the experiment to everyday clinical practice must be demonstrated before starting the study. Thus, financial and ethical limitations are substantial obstacles to conducting in vivo animal studies.

Cyanoacrylate is emerging as an effective agent for permanent embolization, with a low rate of recanalization [10,36,48]. Many studies have established that cyanoacrylate glues have a broad spectrum of indications, ranging from skin-wound closure to the treatment of varicose veins [49,50]. The low viscosity of cyanoacrylate that allows for injection through a microcatheter is among the main features of considerable interest for endovascular applications [17]. Furthermore, the low viscosity allows the embolization of small distal arteries beyond the reach of the microcatheter tip. Moreover, the rapid polymerization of cyanoacrylate results in a smaller radiation dose to the patient when angiographic guidance is used compared to the use of particulate or solid embolization devices, such as plugs or coils.

The effects of cyanoacrylate glues on body tissues have been extensively studied. The deposition of cyanoacrylate within a vessel results in an acute inflammatory reaction in the wall and surrounding tissues. This progresses to a chronic and granulomatous process after approximately one month, with foreign body giant cells and fibrosis. Several studies from this review provided interesting histopathologic considerations that are listed in Table 4.

The main challenge in using cyanoacrylate results from the fast polymerization, which is linked to the multiple reactions involved, including initiation and propagation phases. The low viscosity may increase the risk of reflux with non-target embolization. Finally, cyanoacrylate is radiolucent. In vitro and in vivo studies have been performed to modulate polymerization, for instance, by adding glacial acetic acid [39,43,51], to induce radio-opacity by adding tantalum powder or a contrast agent [26,52], or to decrease vessel adhesivity by adding absolute ethanol [53]. In vivo assessments can help to understand the mechanisms underlying cyanoacrylate polymerization. They are also performed to characterize new liquid embolic agents, such as precipitating hydrophobic injectable liquid (PHIL^®^, Culpan Medical, Victoria, Australia) or ethylene–vinyl alcohol copolymer (EVOH and tantalum, Squid^®^, Emboflu, Gland, Switzerland) [54].

We focused on studies of non-modified arteries, which are more reliable for characterizing polymerization and for designing models of ischemia. Recent studies chiefly used rabbits and swine for both ethical and financial reasons. Swine have interesting resemblances to humans in terms of vessel diameter, hemodynamics, and coagulation [55]. The main drawbacks are the need for dedicated instruments to perform the procedure and the fairly high cost of these large animals. Rabbits are available in most preclinical laboratories and exhibit blood-clotting properties similar to those of humans, although other characteristics show greater differences compared to swine. Rabbit models are less costly and seem to allow medium- to long-term follow-up [43,56]. Canine models are intermediate between swine and rabbits regarding vessel diameter, coagulation properties, and cost. However, the use of dogs has declined over time, probably due to changes in basic science practices. It is worth noting that animal models of non-modified arteries have also been used to test new tools, such as new types of microcatheters [23].

The cyanoacrylate glue most widely used today for endovascular interventions is Histoacryl^®^, i.e., NBCA, with Lipiodol as the radio-opaque agent. This probably explains why most of the recent preclinical studies were completed with Histoacryl^®^. The two countries with the largest number of publications on Histoacryl^®^ identified by our search are the USA and Japan, where this product has been widely available for many years. Bucrylate^®^, or isobutyl 2-cyanoacrylate, was used more often in the 1990s, before studies showed that Histoacryl^®^ (NBCA) had a better safety profile, with notably less inflammation and greater tensile strength [22,57,58]. Glubran^®^ 2, composed of NBCA mixed with the monomer metacryloxysulfolane, was the first cyanoacrylate to receive the EC mark for endovascular use. Although Glubran^®^ 2 is commonly used in Europe for endovascular interventions, it was used in a single animal study identified by our review, with promising results [36].

The tools needed for endovascular interventions using cyanoacrylates vary according to the model used. For instance, narrower introducers are needed for rats and rabbits than for dogs, swine, and primates. Furthermore, the microcatheter, particularly the external diameter of the distal tip, must be appropriate not only for the animal species but also for the embolization site. Our review showed that the renal arteries were most often used in animal studies of cyanoacrylates. Obvious technical reasons explain this preference. Furthermore, the terminal vascularization of the kidneys allows the target embolization of a theoretically restricted area devoid of anastomoses, with better tolerance of the resulting ischemia compared to other organs. These characteristics allow for the monitoring of the vascular reaction over time, which typically consists of acute local inflammation followed by a foreign-body giant-cell reaction and fibrosis [26,59].

## 5. Conclusions

We aimed to provide an exhaustive review of the preclinical animal experiments that used cyanoacrylates for endovascular interventions on non-modified arteries. We assessed the study designs, models, and endovascular procedures performed. Such studies are scarce compared to studies of embolic agents in general, and most of them used Histoacryl^®^ or Bucrylate^®^. Canine, rabbit, and swine models predominated, and the renal arteries were by far the most common embolization site.

Additional work is needed to better understand the mechanism of action of liquid cyanoacrylate glues that are used for endovascular interventions.

## Figures and Tables

**Table 1:** Quantitative data from studies of dog models.

Cyanoacrylate Glue Used	Radiopaque Agent	Other Components	Absolute Cyanoacrylate Concentration (%)	Amount (mL)	Embolization Site	Microcatheter Distal Tip-External Diameter (French)	Introducer Size (French)	Authors	Publication Year
Commercial cyanoacrylate	None	None	0.96	0.05	Superior cerebral artery and anterior communicating artery	DP	DP	Guo et al. [13]	1995
Histoacryl^®^ (NBCA)	Ethiodized oil	Tantalum powder	0.17–0.33	0.5–1	Coronary artery	NR	7.0	Matos et al. [14]	2005
Histoacryl^®^ (NBCA)	Lipiodol	None	0.25	0.1–0.3	Superior mesenteric artery	3.0	5.0	Jae et al. [15]	2008
Bucrylate^®^ (IBCA)	Tantalum powder alone	None	1	0.3–0.5	Left gastric artery, gastroduodenal artery, inferior pancreatico-duodenal artery, and branches of the superior mesenteric artery	3.0	6.7	Dotter et al. [16]	1975
Histoacryl^®^ (NBCA)	Lipiodol	None	0.1–0.5	0.43	Renal artery	2.0	8.0	Takasawa et al. [17]	2012
Bucrylate^®^ (IBCA)	Iophendylate oil	None	0.5	NR	Renal artery	DP	DP	Cromwell et al. [18]	1986
Bucrylate^®^ (IBCA)	Iophendylate oil	Tantalum powder, Glacial acetic acid	0.75	NR	Renal artery and polar arteries	3.6–4.2	7.0	Spiegel et al. [19]	1986
Bucrylate^®^ (IBCA)	Tantalum powder alone	With or without nitrocellulose	0.96–1	4–5.4	Renal artery	3.0	5.0	Sadato et al. [20]	2000
Histoacryl^®^ (NBCA)	Lipiodol, Urogranoic acid	None	0.33–0.7	0.5-2	Renal artery and polar arteries	3.0	DI	Szmigielski et al. [21]	1981
Histoacryl^®^ (NBCA)	Tantalum powder alone	None	0.11–0.26	NR	Renal artery	3.6	NR	Oowaki et al. [22]	2000
Bucrylate^®^ (IBCA)	None	None	1	0.15	Left renal artery	1.5–2.6	NR	Zanetti et al. [8]	1972
Bucrylate^®^ (IBCA)	Iophendylate oil	Tantalum powder	0.75	NR	Branch of renal artery, branch of external carotid artery, and vertebral artery	3.6–4.2	7.0	Debrun et al. [23]	1982
Bucrylate^®^ (IBCA)	Iophendylate oil	None	0.5	NR	Renal and visceral arteries	1.0	6.5	Khangure et al. [24]	1981
Bucrylate^®^ (IBCA)	Iophendylate oil	Tantalum powder	0.75	0.13	Renal artery, splenic artery, and anterior spinal artery via the vertebral artery	3.0	4.1–5.0	ApSimon et al. [25]	1984
Histoacryl^®^ (NBCA)	Iophendylate oil, Lipiodol	None	0.2–0.5	NR	Renal artery, intercostal and lumbar arteries, vertebral arteries, and superior mesenteric artery	NR	5.0	Stoesslein et al. [26]	1982

NR, not reported; DP, direct puncture of the target artery; DI, direct insertion of the microcatheter without using an introducer; IBCA, iso-butyl cyanoacrylate; NBCA, N-butyl-cyanoacrylate.

**Table 2:** Quantitative data from studies of swine models.

Cyanoacrylate Glue Used	Radioopaque Agent	Other Components	Absolute Cyanoacrylate Concentration (%)	Amount (mL)	Embolization Site	Microcatheter Distal Tip-External Diameter (French)	Introducer Size (French)	Authors	Publication Year
Alpha-hexil-cyanoacrylate	Lipiodol	None	0.33	NR	Renal artery and polar arteries (ascending pharyngeal artery and rete mirabile)	2.4	6.0	Izaaryene et al. [27]	2016
Histoacryl^®^ (NBCA)	Lipiodol	None	0.5	NR	Common hepatic artery and internal inguinal artery	2.2	5.0	Tanaka et al. [28]	2015
Histoacryl^®^ (NBCA)	Lipiodol	None	0.5	0.3–0.7	Intercostal artery	2.7	4.0	Hamaguchi et al. [29]	2015
Histoacryl^®^ (NBCA)	Lipiodol	None	0.67	0.5	Superior mesenteric artery or one of its main branches	2.9	6.0	Bruhn et al. [30]	2013
Histoacryl^®^ (NBCA)	Lipiodol	None	0.13–0.5	0.11–0.16	Uterine artery	2.2	4.0	Sonomura et al. [31]	2013
Histoacryl^®^ (NBCA)	Lipiodol	None	0.5–0.1	0.67	Dorsal pancreatic artery	2.1	5.0	Okada et al. [32]	2012
Histoacryl^®^ (NBCA)	Lipiodol	None	0.125	0.2	Bronchial artery	2.5	4.0	Tanaka et al. [33]	2012
Histoacryl^®^ (NBCA)	Lipiodol	None	0.2	0.1–0.4	Sigmoid-rectal branch artery, right colic branch, and middle colic branch	2.5	5.0	Ikoma et al. [34]	2010
Histoacryl^®^ (NBCA)	Lipiodol	None	0.17	NR	Renal and splenic arteries	2.5	5.0	Yonemitsu et al. [35]	2010
Histoacryl^®^ (NBCA)	Lipiodol	None	0.5–0.22	0.2	Renal arterial branches and ascending pharyngeal artery	2.0–2.6	5.0	Levrier et al. [36]	2003
Histoacryl^®^ (NBCA)	Lipiodol	None	0.5	5	Superior mesenteric artery	3.0	8.0	Klein et al. [37]	1996
Avacryl^®^ (NBCA)	Iophendylate oil	None	0.2–0.5	0.2–0.05	Renal artery, hepatic artery, gastro-splenic artery, internal iliac artery, gluteal artery, posterior and anterior deep femoral arteries, popliteal artery, anterior tibial artery, and jejunal artery	3.0	7.0	Widlus et al. [38]	1992
Histoacryl^®^ (NBCA)	Iophendylate oil	Glacial acetic acid	0.50–0.75	0.15	Internal carotid artery (rete mirabile)	4.1	5.0	Brothers et al. [39]	1989
Bucrylate^®^ (IBCA)	Tantalum powder alone	Tantalum powder	0.1	3.3	Internal iliac artery	3.5	7.0	Miller et al. [40]	1978

NR, not reported; IBCA, iso-butyl cyanoacrylate; NBCA, N-butyl-cyanoacrylate.

**Table 3:** Quantitative data from studies of rabbit models.

Cyanoacrylate Glue Used	Radiopaque Agent	Other Components	Absolute Cyanoacrylate Concentration (%)	Amount (mL)	Embolization Site	Microcatheter Distal Tip-External Diameter (French)	Introducer Size (French)	Authors	Publication Year
Histoacryl^®^ (NBCA)	Lipiodol	None	0.5	0.1–0.2	Femoral artery	DP	DP	Wang et al. [41]	2006
Histoacryl^®^ (NBCA)	Iobenzene ester	None	0.5	0.2–0.3	Right carotid artery	2.0–2.7	DI	Shi et al. [42]	2002
Histoacryl^®^ (NBCA)	Lipiodol	Glacial acetic acid	0.2–0.5	NR	Subclavian and femoral arteries	1.8–2.7	4.0	Gounis et al. [43]	2002
Histoacryl^®^ (NBCA)	Ethiodol	Ethiodol	0.5–0.2	0.2	Renal artery	2.7	DI	Sadato et al. [20]	2000
Isostearyl-2-cyanoacrylate (derived from IBCA), Histoacryl^®^ (NBCA)	Lipiodol	None	0.5	0.5	Renal artery	1.5	NR	Oowaki et al. [22]	1999
Bucrylate^®^ (IBCA)	Tantalum powder alone	With or without nitrocellulose	0.96–0.99	0.4–0.8	Renal artery	3.0	DI	Salomonowitz et al. [44]	1983
Histoacryl^®^ (NBCA)	Tantalum powder alone	None	0.11–0.25	NR	Renal artery	3.0–3.6	NR	Günther et al. [45]	1978

NR, not reported; DP, direct puncture of the target artery; DI, direct insertion of the microcatheter without using an introducer; IBCA, iso-butyl cyanoacrylate; NBCA, N-butyl-cyanoacrylate.

**Table 4:** Main histopathologic findings from studies of animal models [3,4,5,6,8,13,14,15,17,27,28,29,32,33,34,35,36,39,40,41,45,46,57,59].

Histopathologic Findings	Minimal Delay between Embolization and Description (Days)	Animal Models
Sponge-like matrix with entrapped erythrocytes in its interstices	1	Swine, dog, rabbit
Desquamation of adjacent to the endothelial cells	1	Swine, dog
Infiltration of neutrophils into the adventitial layer	1	Dog
Infiltration of neutrophils into the intermediate layer	2	Swine, rat
Hyperplasia of adjacent elastic fibrils	7	Dog
Foreign body giant cells adjacent to the polymer	7	Swine, dog, rabbit, rat
Tissue necrosis	7	Swine, dog, rabbit, rat
Infarcted region calcification	21	Rabbit

## Data Availability

All the study data are reported in this article.
